# Reinforcement of Aluminium-Matrix Composites with Glass Fibre by Metallurgical Synthesis

**DOI:** 10.3390/ma13235441

**Published:** 2020-11-29

**Authors:** Małgorzata Zasadzińska, Paweł Strzępek, Andrzej Mamala, Piotr Noga

**Affiliations:** Faculty of Non-Ferrous Metals, AGH University of Science and Technology, 30-059 Kraków, Poland; strzepek@agh.edu.pl (P.S.); amamala@agh.edu.pl (A.M.); pionoga@agh.edu.pl (P.N.)

**Keywords:** aluminium-matrix composites, glass fibre, glass fibre reinforced aluminium matrix composites, metallurgical synthesis, extrusion

## Abstract

Continuous pressure put on researchers all over the world these days to design materials of improved properties create opportunities to study new methods of production in conjunction with entirely new and innovative materials such as alloys or composites. The authors in the current research manufactured aluminium reinforced with glass fibre (GF) using metallurgical synthesis, which is an unconventional and not sufficiently studied method of production. The composites with 1, 2 and 5 wt.% of glass fibre were produced with additional material obtained using consolidation of aluminium powder in extrusion process as reference material with 5 wt.% of glass fibre. All the materials were subjected to series of tests in order to determine their microstructure, density, electrical properties, hardness and susceptibility to plastic working in the compression test. It was found that glass fibre during metallurgical synthesis of aluminium composite partially melted and thus did not reinforce the material as well as during extrusion, which has been observed not only in the scanning electron microscope (SEM) and energy-dispersive X-ray (EDX) analysis but also in the analysis of macroscopic physical and mechanical properties. Based on the analysed samples, it may be stated that electrical conductivity of the samples obtained via metallurgical synthesis is higher than might be estimated on the basis of the rule of mixtures and glass fibre content and concerning the sample with 5 wt.% of GF is higher (32.1 MS/m) than of the reference material obtained in extrusion process (30.6 MS/m). Similar situation has been observed in terms of hardness of the tested samples where a minor increase in hardness was noticeable as the amount of glass fibre increased in the composites obtained by metallurgical synthesis. It is believed to be related to the melting of glass fibre, which reduced the volume fraction of GF containing mainly silicon oxides and their diffusion into the aluminium matrix, thus causing solid solution strengthening.

## 1. Introduction

A growing demand for new functional materials based on light metals exhibiting a combination of properties impossible to obtain with conventional alloys manufactured via classical technologies may be observed nowadays. An important group of such materials are metal based composites that made it possible to attain, among others but not limited to, significantly high tensile strength, high modulus of elasticity, high hardness and abrasion resistance, decent rheological resistance both at ambient and increased temperature, high fatigue resistance combined with low density and low thermal expansion. The current state of knowledge and technology enabled the production of metal matrix composites (MMC), polymer matrix composites (PMC) and ceramic matrix composites (CMC). A substantial drawback in terms of composites manufacturing is their high anisotropy of mechanical properties (considering composites reinforced with unidirectional continuous fibre), negligible susceptibility to plastic working (considering CMC), often limited range of operating temperatures (considering PMC), high sensitivity of obtainable properties to the nonrepeatability of the process, high cost of the components and last, but not least, complicated and expensive manufacturing process. Polymer-based composites reinforced with glass fibre (GF) are the most common presently due to their high mechanical properties [1,2], which might be also obtained when reinforcing metal matrix with carbon fibre [3]. Many studies have been also conducted on reinforcing various matrices with basalt [4,5], fly ash [6], wires [7] or metallic powder [8] and the combinations of the above-mentioned in various configurations. There are also studies on reinforcing metallic matrix with fibre of the same metal such as, for instance, reinforcing tungsten with tungsten fibre [9]. High set of mechanical properties and low weight of the metal-based composites (with the matrix usually formed of aluminium, copper, magnesium, titanium, cobalt or iron and less often of nickel, silver or lead) reinforced with particles or fibre-created opportunities for application among various fields of industry such as automotive, aviation, power industry, construction, etc. However, the manufacturing process of the MMC is quite complicated [10,11,12] and since there are many methods of prospective production of these materials, it makes the issue even more complex. The MMC material may be obtained, among others by adding small sections of reinforcement to the liquid metal or alloy, or infiltrating components in the form of thin wires or nanotubes with liquid metal or alloy [13], plastic working [14,15], powder metallurgy [16,17] or roll-bonding process (sandwich layers) [18]. The current work concerns not only manufacturing processes of the composites based on aluminium matrix reinforced with discontinuous glass fibre randomly oriented among the matrix but also the investigation of the influence of the glass fibre addition to the matrix on basic physical and mechanical properties of the composite obtained by two various methods. Such composites are not commonly used in present-day technology and what is more, the scientific research is relatively limited. However, the analysis of the state of knowledge showed, among others, that the authors in [18] investigated the production of aluminium-based composite using the roll-bonding method and examined the tensile strength and total elongation of the aluminium matrix composite reinforced with E-type glass fibre. The authors have proven that the strength properties of the obtained composites significantly increase along with the increase in the rolling cycles with a simultaneous decrease in the elongation when compared to the base material (aluminium EN AW 1050). According to the authors [19], the main problem concerning the manufacturing of the composites reinforced with glass fibre based on aluminium matrix is poor wettability of both materials in relation to each other. They have attempted to improve the abovementioned phenomenon using the structural modifier such as copper, which made reduction in porosity by several percent to be possible.

Over the last few years, a lot of research has been devoted to aluminium alloys (mostly of 6000 series) reinforced with various nonmetallic materials such as SiC, B_4_C or glass fibre. The authors in [20] investigated the influence of the glass fibre reinforcement (4 and 5 wt.% of GF) on the mechanical properties of samples obtained by the stir casting method. They have shown that in the uniaxial static tensile test, the addition of glass fibre causes a visible increase in yield strength (YS) by approximately 20% with no significant decrease in elongation. In [21], the increase in ultimate tensile strength (UTS) is also shown in the analysed samples up to 6 wt.% of GF. However, concerning the samples with higher reinforcement content, a decrease by 25 MPa (down to 120 MPa) was noted, which corresponded to the UTS value of tested samples with 2 wt.% of GF. Similar relation was not noted when concerning RHN hardness of the same samples, where the measured values increased linearly from 22 to 43 as the reinforcement content increased. These results were confirmed in [22] in the range of 2–6 wt.% of GF. The authors in [23] examined Young’s modulus of the aluminium composites regarding 3–12 wt.% of GF and have stated that up to 6 wt.% of GF, the values increase and then above that, content decrease, which is in compliance with the results of [21,22]. There are many research papers concerning MMC with other than aluminium matrices, such as, for instance, titanium matrix, which was extensively studied in the research papers of Zherebtsov et al. [24,25,26] where the influence of hot deformation on the composites microstructure and mechanical properties was investigated. It was proven that hot deformation improves ductility significantly with no negative influence on the YS. Ti-based composites were also studied in [27,28,29] where the reinforcement used was TiC, and the authors discussed various manufacturing methods such as gas-solid reaction, laser powder and reactive sintering. The authors in [27,28] stated that the hardness and wear resistance increased with the TiC content, and the ductility correspondingly decreased.

Gohari et al. in their extensive papers, concerning polymer composites reinforced with GF and their failure analysis of the internally pressurized domes [30,31], have conducted analytical, numerical and experimental studies. They have adopted Tsai-Wu failure criterion along with linear interpolation in order to interpolate the critical internal pressure. Based on their research, the authors have stated that domes made of polymer composite materials are most vulnerable to failure at inner plies, while the outer ply is the last at which the failure occurs. According to the authors of these works, keeping the geometrical ratio of the dome constant resulted in larger domes to have lower failure pressure. Additionally, they stated that higher stress deformation and localized failure occurred when the rhombic spots in woven were present in the composite. Tashnizi et al. [32] in their research paper have also studied polymer-based composite materials but those reinforced with carbon fibre. They investigated the optimal winding angle in laminated composite pipes subjected to patch loading, which would improve their mechanical strength. The authors stated that choosing the optimal winding angle, which according to their research is 55°, would result in minimum difference between the compression and tensile radial displacements, therefore providing the minimum radial deflection at the crucial point, while 0° was proven to be the least safe winding angle.

Currently glass fibre of A, C, D, E, M, R, S, T, and Z types, which vary in terms of chemical composition, production technology and properties, are commonly used. It is so because glass fibre has less expensive manufacturing process than carbon-based fibre and are also characterised with high set of mechanical properties, elasticity, stiffness and resistance to chemical damage [33,34,35]. The glass fibre may come in the form of continuous or chopped strands, fibre, yarns, cloths and mats or tapes or be completely grounded. Pure aluminium (series 1000)-based composites reinforced with glass fibre are not fully investigated both in terms of manufacturing processes and the influence of the glass fibre content on the properties. Therefore, in the current paper, an attempt was made to combine these two materials using two distinctive manufacturing methods and to determine physical, mechanical and structural properties of the obtained materials.

## 2. Materials and Methods

### 2.1. Metallurgical Synthesis and Extrusion

**Figure 1:**
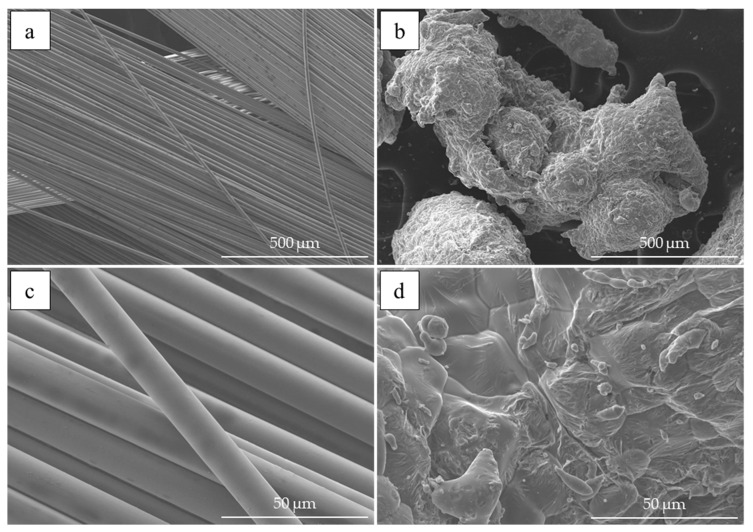
The microscopic images of the input materials used during research; glass fibre (on the left) and aluminium powder (on the right). Scanning electron microscope, magnification: ×100 (**a**,**b**), ×1000 (**c**,**d**)—glass fibre (**a**,**c**) and aluminium powder (**b**,**d**).

Composite materials in the current research were obtained by two distinctive methods i.e., metallurgical synthesis and plastic consolidation of the aluminium powder in the hot extrusion process. Aluminium of 99.7% technical purity (EN AW 1370) in solid form or metallic powder form with a grain size ranging from 400 to 1000 μm was the composite matrix and the reinforcement was E-type glass fibre with 15 μm in diameter, which was cut into equal lengths of about 2 mm. Photographs of the input material (aluminium powder and glass fibre) obtained using scanning electron microscope (Hitachi Ltd., Tokyo, Japan) are presented in Figure 1. The choice of pure aluminium as an input material was made in order to reduce the impact of alloying additives of the alloys on the set of measured electrical and mechanical properties. Thus, the measured changes may be directly attributed to the presence and amount of the fibre reinforcement. When considering the metallurgical synthesis of the composite, it must be noted that it is preferred to use components with just a slight variation in density (at the temperature of the synthesis, the density of glass fibre is equal to approximately 2.5 g/cm^3^ and the density of liquid aluminium is equal to about 2.4 g/cm^3^) in order to reduce the risk of gravitational segregation. The effect of the elevated temperature (corresponding to the temperature of the liquid aluminium) on the glass fibre results in the degradation of its UTS to about 30%–40% of its initial value [36]. Based on the literature knowledge, it is expected that the fibre might partially melt during the process [20,37]. Nevertheless, the expected strength of the reinforcement is still significantly higher than that of the aluminium matrix.

The metallurgical synthesis was carried out in a ceramic crucible using an induction furnace (Termetal, Piekary Śląskie, Poland), which allowed liquid material to be stirred due to electromagnetic field. The solid aluminium was melted in the crucible at 700 °C and previously prepared and preheated to 300 °C glass fibre was placed under the level of liquid metal for 10 s at the amount of 1, 2 and 5 wt.%, thus obtaining a different rate of reinforcement of the aluminium matrix. The proposed GF content has been chosen as the material is being designed to function as carrying-conducting material, which by default is required to meet minimal criteria concerning electrical conductivity. After the stated time, the crucible with the obtained composite was cooled at ambient temperature. The prepared ingots of 40 mm in diameter weighed approximately 75 g.

As reference material, aluminium composite was prepared from aluminium powder with 5 wt.% of GF subjected to initial compaction and further plastic consolidation using a 1 MN concurrent extrusion press (Zakład Mechaniczny Hydromet, Bytom, Poland) equipped with a heated, movable recipient. Initial compaction was carried out using a semiautomatic device for preliminary compaction of loose materials. Input powder material in the amount of 30 g was placed in the cylindrical chamber of the device, where the punch pressed by the piston compressed it with the pressure of 300 kN forming powder metallurgy compact with a diameter of 38 mm and a height of 10 mm. Seven of these compacts constituted for the input material during the extrusion process. The material placed in the recipient was preheated to the temperature of 375 °C for 20 min in order to uniform the temperature throughout its volume prior the extrusion, which was conducted at the speed of 3.7 mm/s on an 8 mm diameter die.

### 2.2. SEM and EDX Observations

Microstructural and energy-dispersive X-ray analysis, which made it possible to determine the presence of the reinforcement and the identification of the chemical composition of the given area was conducted using scanning electron microscope Hitachi SU-70 (Hitachi Ltd., Tokyo, Japan) on previously prepared metallographic specimens taken from the central part of the ingot (concerning samples obtained by the metallurgical synthesis) and at the cross-section of the extruded composite.

### 2.3. Physical Properties

The density of the obtained samples was determined with an analytical scale XA 120/250.4Y (Radwag, Radom, Poland) according to the classical Archimedes method, which uses the measurements of the mass of samples in water and in air, while knowing the liquid and air density at the temperature of the measurement to 1 decimal place. The mean value of density was based on the measurements of 5 different samples.

The electrical conductivity was determined with a SigmaTest 2.069 device (Forester Instruments Inc., Pittsburgh, PA, USA), which is an eddy current device allowing to obtain accurate, nondestructive measurement of electrical conductivity. The measurements are based on the impedance of a complex measuring probe, i.e., the relationship between the voltage drop on the measured impedance and the current flowing in alternating current circuits. The electrical conductivity of the composites was tested at a constant temperature of 20 °C and presented as a mean value of the measurements. Each of the materials obtained in metallurgical synthesis was cut in half with both halves divided into 25 areas of 1 cm^2^ and 5 measurements conducted within each area. The electrical conductivity of the extruded material was determined based on 10 measurements conducted on the samples taken from the beginning, middle and the end of the material.

### 2.4. Mechanical Properties

The next stage of the research was to determine the mechanical properties of the composite materials using Vickers hardness method and the TUKON 2500 hardness tester (Buehler, Lake Bluff, IL, USA) with the load of 5 kgf applied for 10 s. Five measurements on each of the previously mentioned areas were conducted along with 10 measurements on the samples taken from the beginning, middle and the end of the extruded material.

In order to compare the effectiveness of both methods and to verify the influence of the glass fibre reinforcement of the aluminium matrix on the composite deformability while cold working, a compression test was also carried out. The compression tests were performed on a testing machine with a maximum load of 50 kN (Zwick/Roell Group, Ulm, Germany). The samples had a cylindrical shape with height equal to 12 mm and diameter of 8 mm giving the height to diameter ratio of the tested sample in the compression test equal to 1.5:1 [38].

## 3. Results and Discussion

### 3.1. SEM and EDX Observations

**Figure 2:**
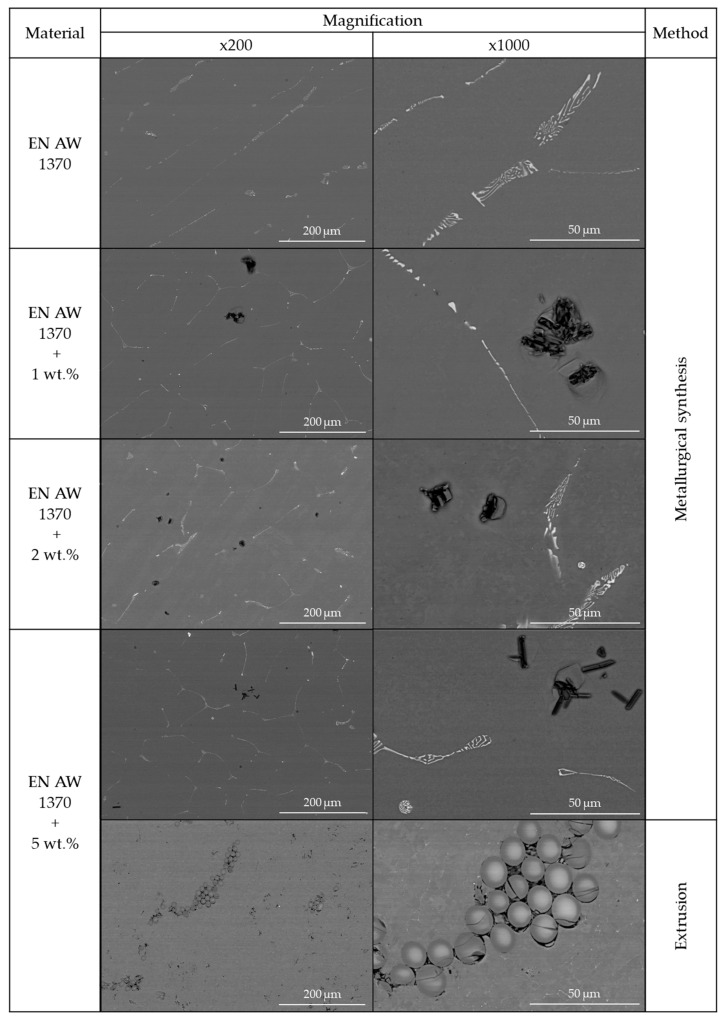
Cross-section of aluminium-glass fibre (Al-GF) composites obtained by metallurgical synthesis and plastic consolidation of metallic powders in extrusion process with various content of GF. Scanning electron microscope, magnification: ×200 and ×1000.

The structural analysis (Figure 2) conducted on the previously prepared metallographic specimens confirmed the presence of glass fibre in the aluminium matrix. Mainly iron precipitations were present regarding pure aluminium (EN AW 1370), which is typical among this aluminium series. In the case of Al-GF composites with various content of glass fibre (1, 2 and 5 wt.%) irregular distribution of the reinforcement in the matrix might be observed in each of the analysed samples, and despite electromagnetic stirring of liquid metal, glass fibre formed irregular conglomerates. Regarding the material obtained by metallurgical synthesis, it was visible that the glass fibre slightly melted due to the influence of elevated temperature during the process, causing a significant reduction in terms of its cross and longitudinal section. This was especially evident in terms of materials with 5 wt.% of GF obtained with two distinctive preparation methods. Regarding plastic consolidation of metallic powder in the extrusion process, the glass fibre remained regular at the cross-section and the process did not affect their shape and dimensions, which was possible due to the lower temperature of the extrusion process (about 375 °C). The forces occurring throughout the process were not sufficient to influence the shape or dimensions of the fibre, however, some of the fibres show minor cracks. Glass fibre also exhibit orientation towards the direction of the applied load in the extrusion process, which may affect the mechanical properties of the composite as examined in [39].

**Figure 3:**
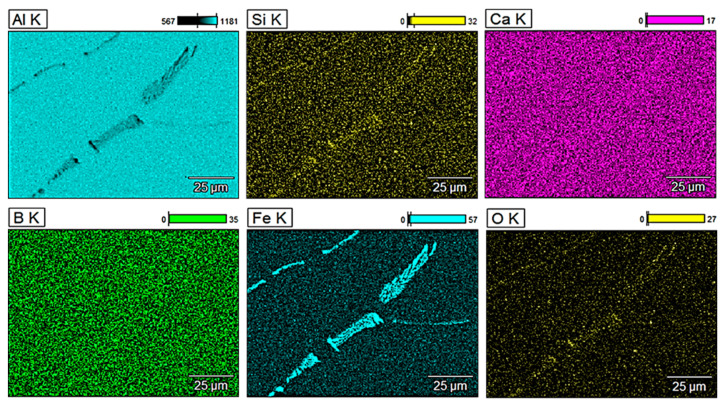
Energy-dispersive X-ray (EDX) mapping of EN AW 1370 (metallurgical synthesis).

**Figure 4:**
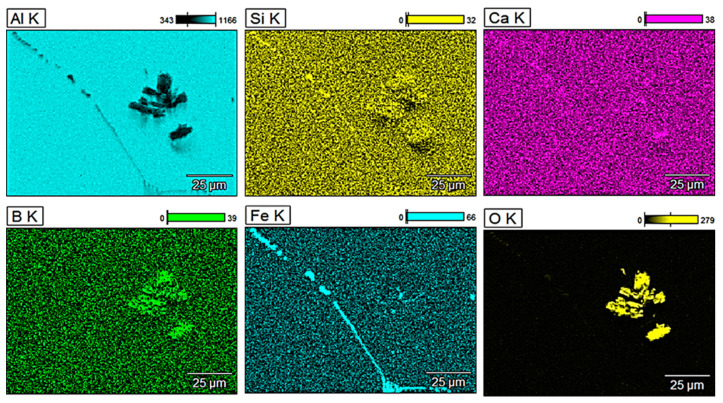
EDX mapping of EN AW 1370 with 1 wt.% of GF reinforcement (metallurgical synthesis).

**Figure 5:**
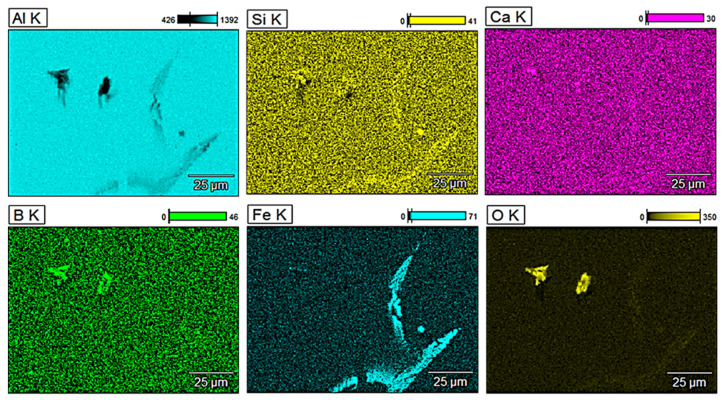
EDX mapping of EN AW 1370 with 2 wt.% of GF reinforcement (metallurgical synthesis).

**Figure 6:**
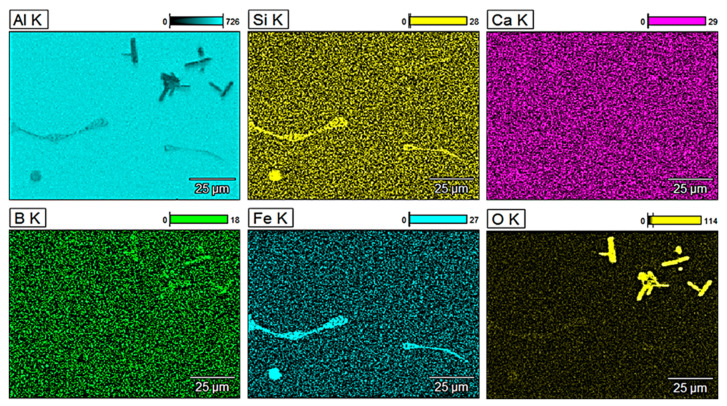
EDX mapping of EN AW 1370 with 5 wt.% of GF reinforcement (metallurgical synthesis).

For a more detailed analysis of the glass fibre reinforcement, observations of the microstructure were performed along with the analysis of the chemical composition of selected areas. Figure 3 shows energy-dispersive X-ray (EDX) mappings of pure aluminium obtained by metallurgical synthesis and Figure 4, Figure 5 and Figure 6 show EDX mappings of given areas of samples containing glass fibre obtained by metallurgical synthesis.

EDX analysis of the EN AW 1370 base material confirmed the presence of typical for this series of aluminium iron precipitations, there was, however, no silicon, calcium, boron, or other oxides present in the matrix.

Regarding composite material with 1 wt.% of GF, the presence of oxides, especially boron and silicon oxides, was clearly visible as concerning the mappings of Si (yellow) and B (green), the concentration of yellow and green points, respectively, is much higher at the area where oxygen and thus oxides were detected. It has been similarly confirmed in terms of 2 and 5 wt.% of GF. As before, typical iron precipitation was also present.

**Figure 7:**
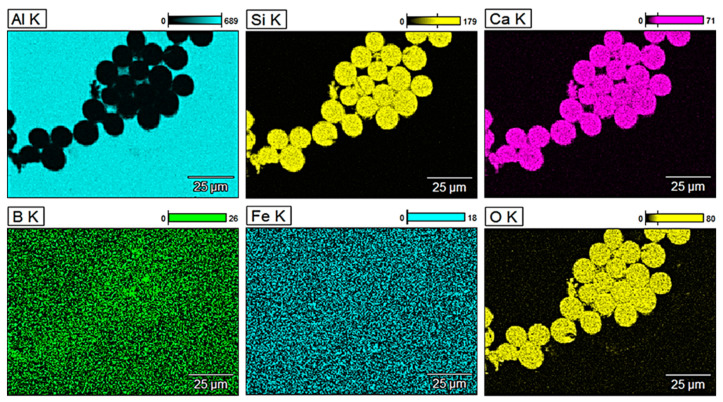
EDX mapping of EN AW 1370 with 5 wt.% of GF reinforcement (extrusion process).

Iron precipitations as well as boron and silicon oxides were also visible in terms of the composite material with 2 wt.% of GF. Since the base material is aluminium EN AW 1370, it consists of roughly 99.7 wt.% of Al, 0.2 wt.% of Fe and 0.1 wt.% of Si, which explains the primary precipitates of Al-Fe (Si). These are visible at Fe mappings of all the tested samples obtained by metallurgical synthesis and are not visible concerning sample obtained in the extrusion process (Figure 7).

Regarding material with the highest GF content (5 wt.%) oxides are again clearly visible. The presence of calcium was observed to some extent in the entire volume of the matrix. Similarly, the amount of detected silicon in the glass fibre was very limited, however, again, it was present in the entire analysed area, including the matrix, which was not the case in terms of pure EN AW 1370 material. Material with 5 wt.% of GF obtained by extrusion process was also analysed (Figure 7), and significantly different values and distribution of silicon and calcium were observed. The reinforcement shows mainly silicon oxides, which form about 50% of glass fibre’ chemical composition. Additionally, a substantial amount of Ca oxides was observed, which was not the case in any of the materials obtained by metallurgical synthesis. A clear presence of these elements in the reinforcement of the extruded material may indicate that during metallurgical synthesis, glass fibre partially melted causing Si and Ca to merge with the aluminium matrix. It might also be confirmed by the fact that in the above-presented EDX mappings, the presence of these elements was observed to some extent in the entire volume of the studied areas, unlike the extruded material where they were present only in the fibre.

### 3.2. Physical Properties

The obtained aluminium composites reinforced with glass fibre were also subjected to fundamental tests in order to determine their physical and mechanical properties. They are presented collectively in Table 1.

The tests performed to determine the density of the samples showed that pure material, without the addition of fibre, had the highest density, and the glass fibre density was the lowest and equal to approximately 2.55 g/cm^3^. Throughout the conducted research, it was proven that despite only a slight difference in the density between both input materials, the composites obtained by metallurgical synthesis showed lower density values as glass fibre reinforcement content increased. The material obtained by plastic consolidation of powders in extrusion process had higher density value than the material with 5 wt.% of GF obtained by metallurgical synthesis. The decrease in density was greater than it may seem from the rule of mixtures, which suggests the presence of micro-defects disturbing the coherence of the material such as the voids between the fibre in the case of an extruded material (Figure 2) or microporosity in the case of composites obtained by metallurgical synthesis. It should be borne in mind that during metallurgical synthesis (based on the conducted EDX analysis), glass fibre partially melted causing the reinforcement content to be lower than assumed, while dissolved components diffused into the metal matrix changing its density.

The electrical properties tests of the obtained composites showed that the presence of the glass fibre reinforcement decreased the electrical conductivity, and the decrease was directly proportional to the increase in the reinforcement content. Regarding materials obtained by metallurgical synthesis, the measured electrical conductivity was higher than might be estimated based on the rule of mixtures and glass fibre content. An argument was proposed that it was related to the melting of glass fibre, which reduced the volume fraction of GF resulting in higher electrical conductivity, whereas at the same time, the elements, which were derived from the fibre, dissolved into the matrix and degraded its electrical conductivity, therefore, the observed effect was the result of synergy of the above-mentioned reasons. The lowest value of electrical conductivity was measured for the extruded material, which might be resulted from the greater share of GF at the cross-section of the composite in comparison to the materials obtained by metallurgical synthesis. Partially melted during metallurgical synthesis, glass fibre, which diffused into aluminium matrix, reduced its share at the cross-section and directly lowered the amount of obstacles limiting the electrically active cross-section and free flow of the electrons.

### 3.3. Mechanical Properties

**Figure 8:**
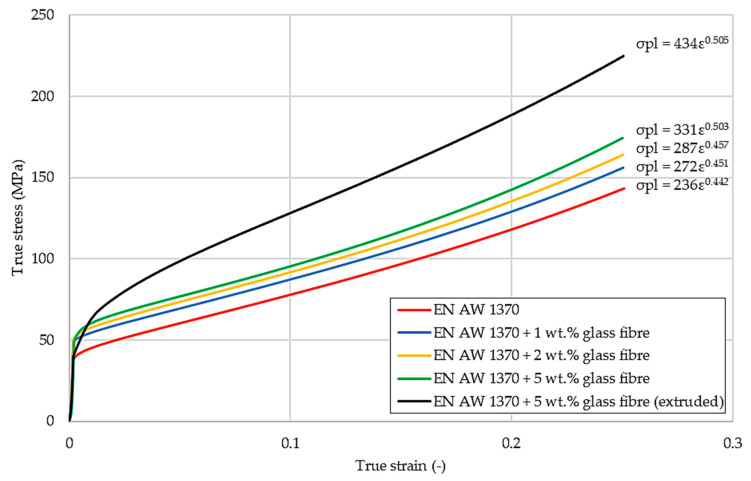
Compression characteristics of Al-GF composites.

The analysis of the hardness results obtained using Vickers method showed that the hardness values increased with the increase in the glass fibre content, which is in agreement with the results obtained by the authors in [40] where the studied composites comprised aluminium series 6061 and glass fibre content from 3 to 12 wt.%. The composite obtained by the extrusion processes exhibits higher mechanical properties when compared to the material obtained by the metallurgical synthesis, which might be related to the fact that the extruded material was subjected to much higher stresses causing strain hardening and explaining the higher values of hardness. A minor increase in hardness was noticeable as the amount of glass fibre increased in the composites obtained by metallurgical synthesis, which could be linked to the partial melting of GF containing mainly silicon oxides and their diffusion into the aluminium matrix, thus causing solid solution strengthening. However, the hardness test did not fully show the influence of glass fibre reinforcement of the metal matrix on the mechanical properties. Therefore, a uniaxial static compression test was carried out, which would allow to determine the plastic deformability of the composite. Figure 8 shows the compression characteristics of tested Al-GF composites.

The conducted compression tests showed that the materials obtained by metallurgical synthesis had similar course of the compression true stress–true strain curves. As expected, pure aluminium (EN AW 1370) had the lowest compressive strength without losing coherence of the material. The plastic resistance of the tested samples rises as the amount of the reinforcement increases. The plastic resistance of the sample obtained in the extrusion process was noticeably higher when compared to the samples obtained by metallurgical synthesis and the difference was equal to about 45 MPa in the case of samples with 5 wt.% of GF. It might be explained by the temperatures of the conducted processes as the decrease in tensile strength of E-glass fibre when subjected to elevated temperatures was proved in [41]. The strain-hardening coefficients have also been defined and attached at the diagram (Figure 8). There are many models describing the hardening of the material concerning its plastic working, for instance, using the aforementioned coefficients K and n, where K is the strength coefficient and n is the strain hardening exponent. The values of these coefficients for all of the tested materials are collectively presented in Table 2. According to the known literature [42], the strain hardening exponent must be in the range between 0 and 1, where 0 means an ideally plastic body and 1 means ideally elastic body.

## 4. Conclusions

Materials obtained by both metallurgical synthesis and plastic consolidation of metallic powder in extrusion process showed the presence of glass fibre in the aluminium matrix, which confirmed the possibility of obtaining the reinforcement effect using both of these methods. However, during the metallurgical synthesis, the fibre partially melted, which was confirmed in the EDX analysis, and thus, the mechanical properties of the composite were lower than might be expected. The increase in the proportion of glass fibre reinforcement in the composite lead to a decrease in density and electrical conductivity with a simultaneous increase in hardness. However, regardless of the production method, electrical conductivity remained above the value of 30 MS/m and was slightly lower (30.6 MS/m) concerning the extruded sample than the sample with the same amount of glass fibre obtained by metallurgical synthesis (32.1 MS/m). Electrical conductivity of the tested samples obtained by metallurgical synthesis is higher than might be estimated on the basis of the rule of mixtures and glass fibre content. Concerning the hardness of the tested samples, a minor increase in hardness has been noted (19.9 HV5 for pure Al, 21.3 HV5 with 1 wt.% of GF, 22.6 HV5 with 2 wt.% of GF and 24.1 HV5 with 5 wt.% of GF) as the amount of glass fibre increased. It is believed to be the effect of partial melting of glass fibre and, thus, reduction in the volume fraction of glass fibre containing mainly silicon oxides and their diffusion into the aluminium matrix, resulting in solid solution strengthening. The final verification of the mechanical properties in the compression test, which is also the assessment of deformability, showed that the material with the same glass fibre content (5 wt.%) obtained by metallurgical synthesis showed lower compressive strength without brittle cracking.

## Figures and Tables

**Table 1:** Physical and mechanical properties of aluminium-glass fibre (Al-GF) composites with various reinforcement content obtained by various methods of production.

Method	Metallurgical Synthesis			Extrusion	
Material	EN AW 1370	EN AW 1370 + 1 wt.%	EN AW 1370 + 2 wt.%	EN AW 1370 + 5 wt.%	
Density (g/cm^3^)	2.7	2.66	2.64	2.61	2.64
Electrical conductivity (MS/m)	35.2	34.8	34.5	32.1	30.6
Hardness (HV5)	19.9	21.3	22.6	24.1	30

**Table 2:** Strain-hardening coefficients of the tested samples derived from compression true stress–true strain curves.

Material	K (MPa)	n
EN AW 1370	236	0.442
EN AW 1370 + 1 wt.% GF	272	0.451
EN AW 1370 + 2 wt.% GF	287	0.457
EN AW 1370 + 5 wt.% GF	331	0.503
EN AW 1370 + 5 wt.% GF (extruded)	434	0.505

## References

[1] Gnanavelbabu A., Saravanan P., Rajkumar K., Sabarinathan P., Karthikeyan S. (2018). Mechanical strengthening effect by various forms and orientation of glass fibre reinforced isopthalic polyester polymer composite. Mater. Today Proc..

[2] Chawla K.K. (2012). Composite Materials.

[3] Hou L., Wu R., Wang X., Zhang J., Zhang M., Dong A., Sun B. (2017). Microstructure, mechanical properties and thermal conductivity of the short carbon fiber reinforced magnesium matrix composites. J. Alloys Compd..

[4] Brainard Abraham C., Boobesh Nathan V., Rajesh Jaipaul S., Nijesh D., Manoj M., Navaneeth S. (2020). Basalt fibre reinforced aluminium matrix composites—A review. Mater. Today.

[5] Vannan E., Vizhian S.P. (2014). Microstructure and mechanical properties of as cast aluminium alloy 7075/basalt dispersed metal matrix composites. J. Miner. Mater. Charact. Eng..

[6] Patel S., Rana R., Singh S.K. (2017). Study on mechanical properties of environment friendly Aluminium E-waste composite with fly ash and e-glass fiber. Mater. Today Proc..

[7] Raja R., Jannet S., George L., Rao V.M., Sebastian N., Thomas S.P. (2020). Synthesis of Al and Cu wires embedded glass fiber reinforced polymer (AWGFRP and CWGFRP) composites and their mechanical characterization. Mater. Today Proc..

[8] Gao H., Liu X., Zhang S., Qi J. (2018). Synergistic effect of glass fibre and Al powder on the mechanical properties of glass-ceramics. Ceram. Int..

[9] Zhang L., Jiang Y., Fang Q., Liu R., Xie Z., Zhang T., Wang X., Liu C. (2017). Comparative investigation of tungsten fibre nets reinforced tungsten composite fabricated by three different methods. Metals.

[10] Arunkumar S., Sundaram M.S., Kanna K.S., Vigneshwara S. (2020). A review on aluminium matrix composite with various reinforcement particles and their behaviour. Mater. Today Proc..

[11] Arab S.M., Karimi S., Jahromi S.A.J., Javadpour S., Zebarjad S.M. (2015). Fabrication of novel fiber reinforced aluminum composites by friction stir processing. Mater. Sci. Eng. A.

[12] Samal P., Vundavilli P.R., Meher A., Mahapatra M.M. (2020). Recent progress in aluminum metal matrix composites: A review on processing, mechanical and wear properties. J. Manuf. Process..

[13] Knych T., Kwaśniewski P., Kiesiewicz G., Mamala A., Kawecki A., Smyrak B. (2014). Characterization of nanocarbon copper composites manufactured in metallurgical synthesis process. Met. Mater. Trans. B.

[14] Ramesh C.S., Hirianiah A.K., Harishanad S., Prakash Noronha N. (2012). A review on hot extrusion of Metal Matrix Composites (MMC’s). Int. J. Eng. Sci..

[15] Reynolds N., Awang-Ngah S., Williams G., Hughes D.J. (2020). Direct processing of structural thermoplastic composites using rapid isothermal stamp forming. Appl. Compos. Mater..

[16] Bhuyan P., Singh H., Kumar L., Sharma N., Panda D., Verma D., NasmulAlam S. (2016). Development of Cu-E-glass fiber composites by powder metallurgy route. IOP Conf. Ser. Mater. Sci. Eng..

[17] Kumar K.R. (2020). Characterization, mechanical and wear behaviour of magnesium (AZ91D)/graphite/tungsten carbide hybrid composites fabricated by powder metallurgy. Trans. Indian Inst. Met..

[18] Alizadeh M., Shakery A., Salahinejad E. (2019). Aluminum-matrix composites reinforced with E-glass fibers by cross accumulative roll bonding process. J. Alloys Compd..

[19] Ertürk A., Aydın I. (2017). Enhanced mechanical performance of aluminum glass fiber reinforced foam material by cu modification. Acta Phys. Pol. A.

[20] Subramani N., Ganesh M.J. (2019). Characteristics of A6061/(Glass Fibre + AL_2_O_3_ + SiC + B_4_C) Reinforced Hybrid Composite Prepared through STIR Casting. Adv. Mater. Sci. Eng..

[21] Vinayashree, Shobha R. (2018). Study on mechanical property of aluminium 6061 with E glass fiber reinforced composite. Appl. Mech. Mater..

[22] Jayaprakash H.V. (2018). Studies on the mechanical properties of aluminium 6061 glass fiber composite materials. Int. J. Sci. Basic Appl. Res..

[23] Gouse M., Reddy B. (2017). Ambadas. An experimental study on mechanical properties of eglass fiber reinforced AL6061 alloy MMCs. J. Emerg. Technol. Innov. Res.

[24] Zherebtsov S., Ozerov M., Klimova M., Stepanov N., Vershinina T., Ivanisenko Y., Salishchev G. (2018). Effect of high-pressure torsion on structure and properties of Ti-15Mo/TiB metal-matrix composite. Materials.

[25] Zherebtsov S., Ozerov M., Povolyaeva E., Sokolovsky V., Stepanov N., Moskovskikh D.O., Salishchev G. (2019). Effect of hot rolling on the microstructure and mechanical properties of a Ti-15Mo/TiB metal-matrix composite. Metals.

[26] Zherebtsov S., Ozerov M., Klimova M., Moskovskikh D.O., Stepanov N., Salishchev G. (2019). Mechanical behavior and microstructure evolution of a Ti-15Mo/TiB titanium—Matrix composite during hot deformation. Metals.

[27] Zhang C., Guo Z., Yang F., Wang H., Shao Y., Lu B. (2018). In situ formation of low interstitials Ti-TiC composites by gas-solid reaction. J. Alloys Compd..

[28] Zhang Y., Wei Z., Shi L., Xi M. (2008). Characterization of laser powder deposited Ti-TiC composites and functional gradient materials. J. Mater. Process. Technol..

[29] Zhao H., Cheng Y.-B. (1999). Formation of TiB2 ± TiC composites by reactive sintering. Ceram. Int..

[30] Gohari S., Sharifi S., Vrcelj Z., Yahya M.Y. (2015). First-ply failure prediction of an unsymmetrical laminated ellipsoidal woven GFRP composite shell with incorporated surface-bounded sensors and internally pressurized. Compos. Part B Eng..

[31] Gohari S., Sharifi S., Burvill C., Mouloodi S., Izadifar M., Thissen P. (2019). Localized failure analysis of internally pressurized laminated ellipsoidal woven GFRP composite domes: Analytical, numerical, and experimental studies. Arch. Civ. Mech. Eng..

[32] Tashnizi E.S., Gohari S., Sharifi S., Burvill C. (2020). Optimal winding angle in laminated CFRP composite pipes subjected to patch loading: Analytical study and experimental validation. Int. J. Press. Vessel. Pip..

[33] Wallenberger F.T., Watson J.C., Li H. (2001). Glass Fibers. ASM Handbook.

[34] Bahadur S., Zheng Y. (1990). Mechanical and tribological behavior of polyester reinforced with short glass fibers. Wear.

[35] Hearle J.W.S. (2001). High-Performance Fibre.

[36] Thomason J.L., Jenkins P.G., Yang L. (2016). Glass Fibre Strength—A Review with Relation to Composite Recycling. Fibers.

[37] Samanta A., Ding H., Ding H. (2018). A novel selective laser melting process for glass fiber-reinforced metal matrix composites. Manuf. Lett..

[38] (2011). ISO 13314:2011 Mechanical Testing of Metals-Ductility Testing-Compression Test for Porous and Cellular Metals.

[39] Ekşı S., Genel K. (2017). Comparison of mechanical properties of unidirectional and woven carbon, glass and aramid fiber reinforced epoxy composites. Acta Phys. Pol. A.

[40] Kumar M., Shankar U. (2012). Evaluation of mechanical properties of aluminum alloy 6061—Glass particulates reinforced metal matrix composites. Int. J. Mod. Eng. Res. Technol..

[41] Boczkowska A., Kapuścinski J., Puciłowski K., Wojciechowski S. (2000). Kompozyty.

[42] Hollomon J.H. (1945). Tensile deformation. Trans. Metall. Soc. AIME.

